# Supplementary material for: Gut Microbiome-Targeted Modulations Regulate Metabolic Profiles and Alleviate Altitude-Related Cardiac Hypertrophy in Rats
Source: Microbiol Spectr. 2022 Feb 9;10(1):e01053-21. doi: 10.1128/spectrum.01053-21 (PMC8826942; doi:10.1128/spectrum.01053-21)
Supplement: SUPPLEMENTAL FILE 1 — Supplemental material. Download SPECTRUM01053-21_Supp_1_seq9.pdf, PDF file, 1.9 MB [file spectrum01053-21_supp_1_seq9.pdf]

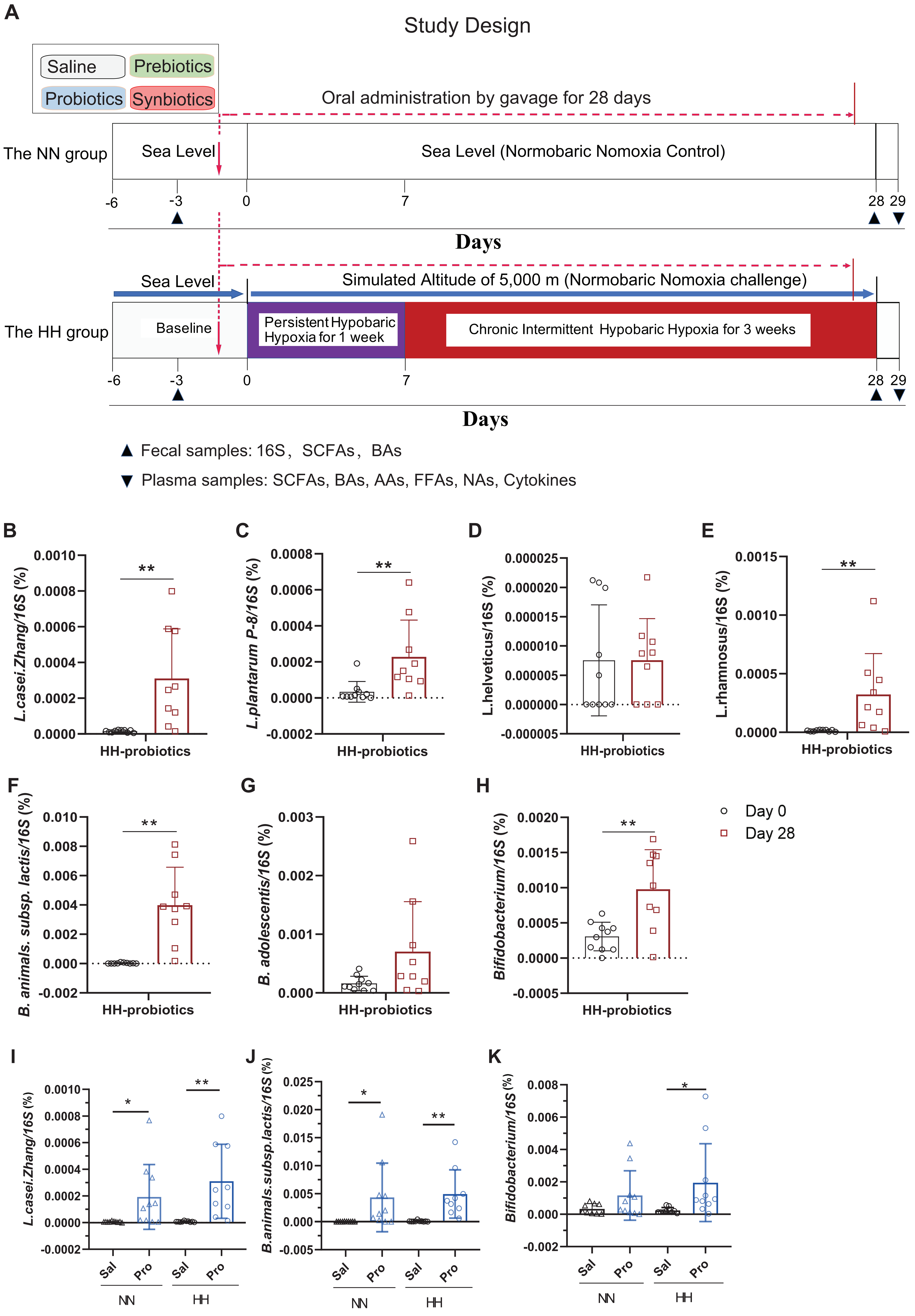

Figure S1. Study design and probiotics detection. A After a 6-days adaptation period, rats were randomly divided into normobaric normoxia (NN) and hypobaric hypoxia (HH) groups. NN rats were kept at sea level. HH rats were placed in a hypobaric chamber simulating 5,000m high altitude environment. Rats in each environment were further divided into 4 groups, gavaged with saline, probiotics, prebiotics and synbiotics for continuous 28 days. Fecal samples were collected at baseline and end of the experiment. Plasma samples and heart tissues were sampled on day 29. **B-H** The levels of L. casei Zhang (**B**), L. plantarum P-8 (**C**), L. helveticus (**D**), L. rhamnosus (**E**), B. animals sub. lactis (**F**), B. adolescentis (**G**) and Bifidobacterium (**H**) on day 0 and day 28 in HH-probiotics group. **I-J** The levels of L. casei Zhang (**I**), B. animals sub. lactis (**J**) and Bifidobacterium (**K**) in both saline and probiotics groups on day 28. Data are presented as means ± S.D.. Statistical significance was performed by GraphPad Prism 8.0 software using t-test. *, P < 0.05; **, P < 0.01.

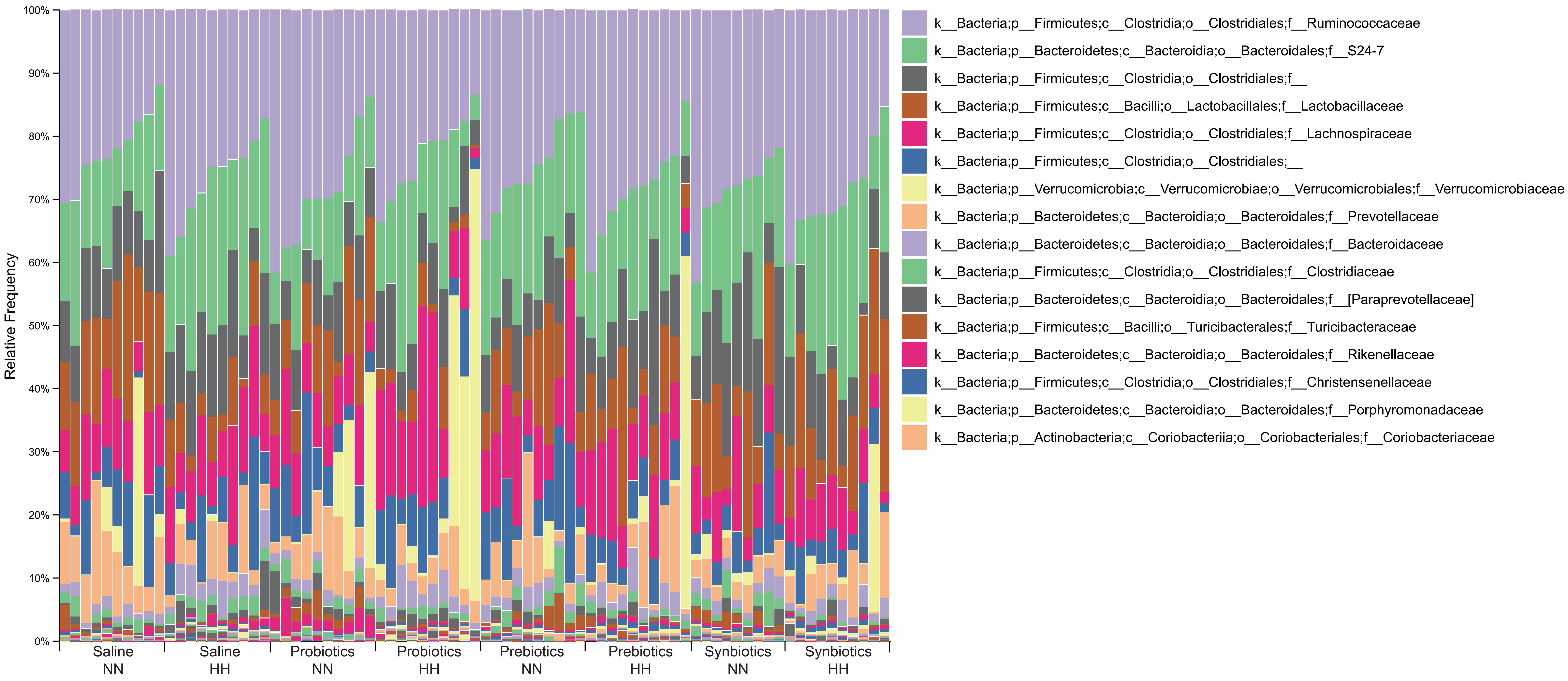

Figure S2. The taxonomy composition of gut microbiome on the family level on day 28.

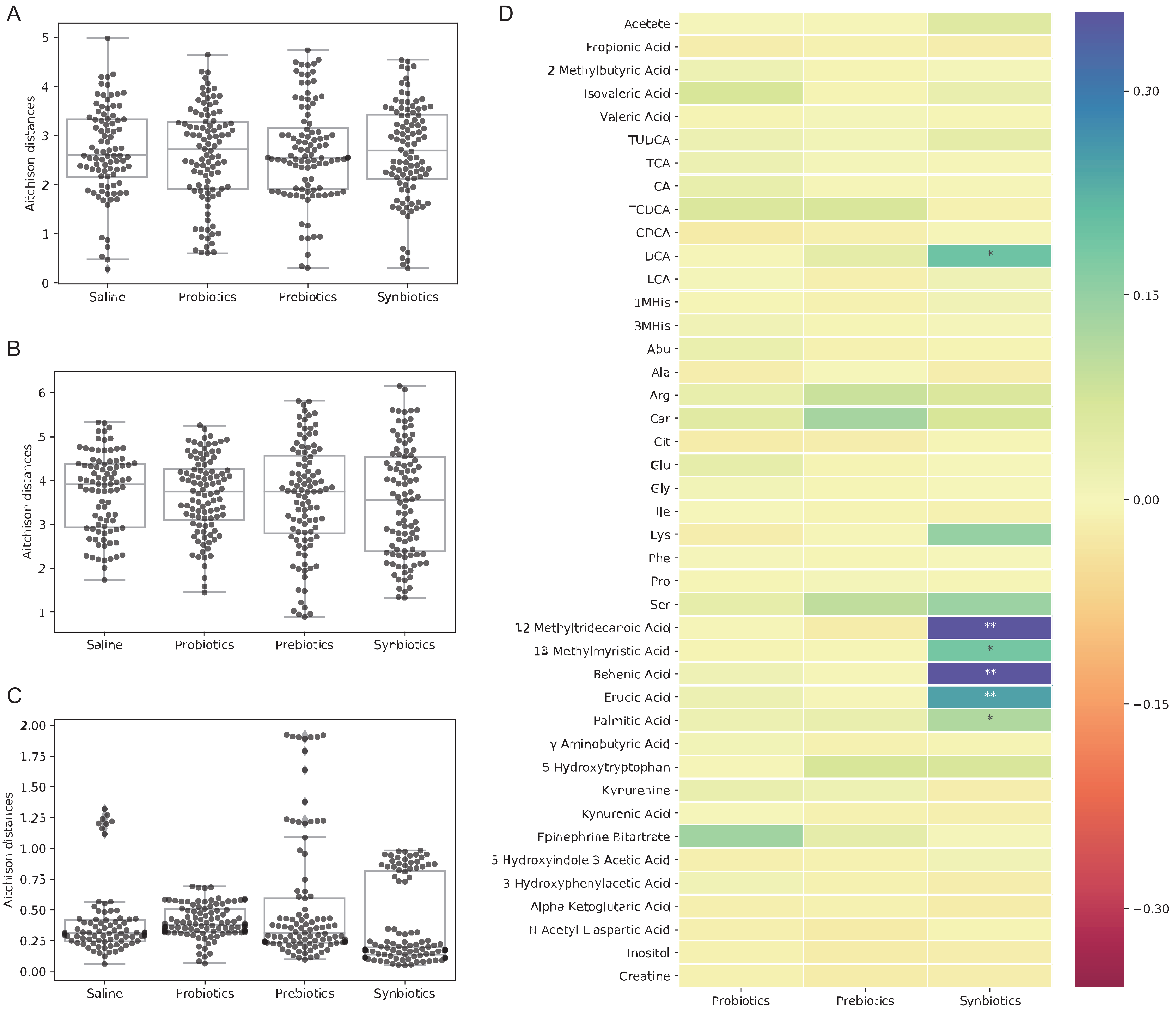

Figure S3. Effect of treatments on plasma metabolome and cytokines. A-C Aitchison distances between NN and HH samples in each treatment remained unchanged compared with saline group in SCFAs (**A**), BAs (**B**) and FFAs (**C**). **D** Heatmap of treatment effects on the plasma metabolites that were statistically significantly altered by hypobaric hypoxia exposure. The color indicates the effect size of the treatments on hypobaric hypoxia computed from two-way ANOVA. Stars indicate significant interaction, *FDR < 0.05, **FDR < 0.01.

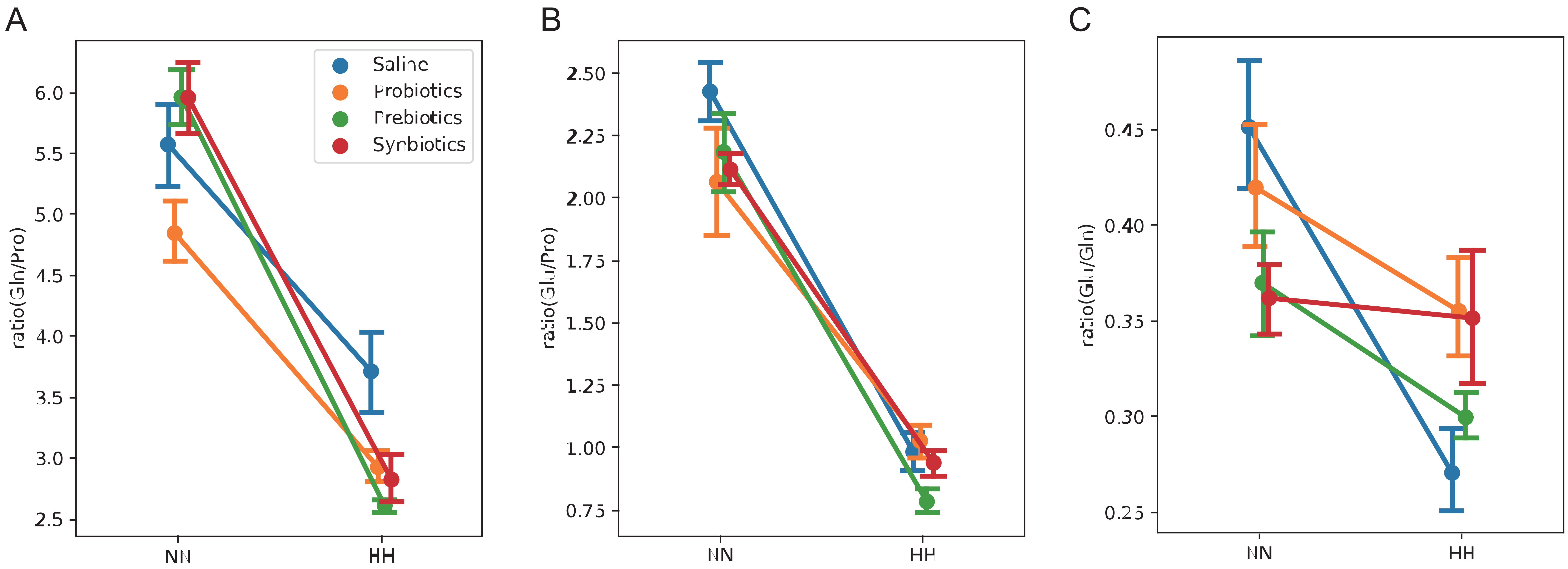

Figure S4. The ratio of Gln/Pro, Glu/Pro and Glu/Gln.

Table S1. Permutation Multivariate Analysis of Variance (PERMANOVA) analysis between every two treatments in NN or HH environment.

NN/HH	treatment1	treatment2	sample size	test statistic	p-value
NN	Saline	Probiotics	20	1.574137324	0.001
NN	Saline	Prebiotics	20	1.597147607	0.001
NN	Saline	Synbiotics	19	1.53715751	0.001
NN	Probiotics	Prebiotics	20	1.77631957	0.001
NN	Probiotics	Synbiotics	19	1.617509012	0.001
NN	Prebiotics	Synbiotics	19	1.448782649	0.001
HH	Saline	Probiotics	20	1.533193013	0.001
HH	Saline	Prebiotics	20	1.534971149	0.001
HH	Saline	Synbiotics	20	1.805968577	0.001
HH	Probiotics	Prebiotics	20	1.657886022	0.001
HH	Probiotics	Synbiotics	20	1.566360846	0.001
HH	Prebiotics	Synbiotics	20	1.471189248	0.001

Table S2. Information of differential ASVs.

ASV	taxonomy
ASV_1	k__Bacteria; p__Firmicutes; c__Clostridia; o__Clostridiales; f__Ruminococcaceae; g__Oscillospira; s__
ASV_2	k__Bacteria; p__Firmicutes; c__Bacilli; o__Lactobacillales; f__Streptococcaceae; g__Lactococcus; s__garvieae
ASV_3	k__Bacteria; p__Firmicutes; c__Clostridia; o__Clostridiales; f__Ruminococcaceae; g__; s__
ASV_4	k__Bacteria; p__Firmicutes; c__Clostridia; o__Clostridiales; f__Ruminococcaceae; g__Ruminococcus; s__
ASV_5	k__Bacteria; p__Bacteroidetes; c__Bacteroidia; o__Bacteroidales; f__Rikenellaceae; g__Alistipes; s__onderdonkii
ASV_6	k__Bacteria; p__Firmicutes; c__Clostridia; o__Clostridiales; f__; g__; s__
ASV_7	k__Bacteria; p__Firmicutes; c__Clostridia; o__Clostridiales; f__Lachnospiraceae; g__Coprococcus; s__
ASV_8	k__Bacteria; p__Firmicutes; c__Clostridia; o__Clostridiales; f__Lachnospiraceae
ASV_9	k__Bacteria; p__Firmicutes; c__Clostridia; o__Clostridiales; f__Ruminococcaceae; g__; s__
ASV_10	k__Bacteria; p__Firmicutes; c__Clostridia; o__Clostridiales; f__Ruminococcaceae; g__Oscillospira; s__
ASV_11	k__Bacteria; p__Firmicutes; c__Clostridia; o__Clostridiales; f__Lachnospiraceae
ASV_12	k__Bacteria; p__Actinobacteria; c__Coriobacteriia; o__Coriobacteriales; f__Coriobacteriaceae; g__Adlercreutzia; s__
ASV_13	k__Bacteria; p__Firmicutes; c__Clostridia; o__Clostridiales
ASV_14	k__Bacteria; p__Firmicutes; c__Clostridia; o__Clostridiales; f__Ruminococcaceae; g__; s__
ASV_15	k__Bacteria; p__Firmicutes; c__Clostridia; o__Clostridiales; f__Lachnospiraceae; g__; s__
ASV_16	k__Bacteria; p__Firmicutes; c__Clostridia; o__Clostridiales; f__Lachnospiraceae; g__; s__
ASV_17	k__Bacteria; p__Firmicutes; c__Clostridia; o__Clostridiales; f__Ruminococcaceae
ASV_18	k__Bacteria; p__Firmicutes; c__Clostridia; o__Clostridiales; f__Ruminococcaceae; g__Ruminococcus; s__bromii
ASV_19	k__Bacteria; p__Firmicutes; c__Clostridia; o__Clostridiales; f__; g__; s__
ASV_20	k__Bacteria; p__Bacteroidetes; c__Bacteroidia; o__Bacteroidales; f__S24-7; g__; s__

Table S3. qPCR primers.

No.	Targets	Specificity	Direction Sequence (Forward)	Direction Sequence (Reverse)	References
1	*L. casei Zhang*	Strain	CCGACGTACCAGCTCACT	TGAGCCGCTATCTGATAGTCTT	[PMID: 30409169]
2	*L. plantarum P-8*	Strain	ACTAACGGGAGGAGTGAT	ATAGTTCTCAAATCGGGAC	[PMID: 29867805]
3	*L. rhamnosus*	Species	GCCGATCGTTGACGTTAGTTGG	CAGCGGTTATGCGATGCGAAT	[PMID: 32295530]
4	*L. helveticus*	Species	CTACTTCGCAGGCGTTAACT	GTACTTGATGCTCGCATACC	[PMID: 32295530]
5	*B. animalis subsp. lactis*	Species	GTGGAGACACGGTTTCCC	CACACCACACAATCCAATAC	[PMID: 14503690]
6	*B. adolescentis*	Species	CTCCAGTTGGATGCATGTC	CGAAGGCTTGCTCCCAGT	[PMID: 14503690]
7	*Bifidobacterium*	Genus	CTCCTGGAAACGGGTGG	GGTGTTCTTCCCGATATCTACA	[PMID: 14503690]
8	16S rRNA Variable Region 3	Bacteria	CCTACGGGAGGCAGCAG	GTATTACCGCGGCTGCTGG	[PMID: 30273610]
9	Natriuretic peptide A (ANP)		GTGTCCAACACAGATCTGATGG	GCCAGCGAGCAGAGCCCTCA	
10	Natriuretic peptide B (BNP)		TGGGAAGTCCTAGCCAGTCTC	GCCGATCCGGTCTATCTTCTG	
11	Collagen type I		ATCAGCCCAAACCCCAAGGAGA	CGCAGGAAGGTCAGCTGGATAG	
12	Collagen type III		TGATGGGATCCAATGAGGGAGA	GAGTCTCATGGCCTTGCGTGTTT	
13	Cardiac myosin heavy chain alpha isoform (αMHC)		GCCCTTTGACATCCGCACAGAGT	TCTGCTGCATCACCTGGTCCTCC	
14	Cardiac myosin heavy chain beta isoform (βMHC)		GCGGACATTGCCGAGTCCCAG	GCTCCAGGTCTCAGGGCTTCACA	
15	Glyceraldehyde-3-phosphate dehydrogenase (*Gapdh*)		GCATCCTGCACCACCAACTG	CACAGTCTTCTGAGTGGCAG	
